# Effects of −10° and −30° head-down tilt on cerebral blood velocity, dynamic cerebral autoregulation, and noninvasively estimated intracranial pressure

**DOI:** 10.1152/japplphysiol.00283.2021

**Published:** 2022-02-24

**Authors:** Tomokazu Kato, Takuya Kurazumi, Toru Konishi, Chiharu Takko, Yojiro Ogawa, Ken-ichi Iwasaki

**Affiliations:** ^1^Division of Hygiene, Department of Social Medicine, Nihon University School of Medicine, Tokyo, Japan; ^2^Institute for Exercise and Environmental Medicine, Texas Health Presbyterian Hospital Dallas, Dallas, Texas; ^3^Department of Neurology, University of Texas Southwestern Medical Center, Dallas, Texas; ^4^Air Staff Office, Japan Air Self-Defense Force, Tokyo, Japan

**Keywords:** cephalad fluid shift, cerebral circulation, transcranial Doppler sonography, transfer function analysis

## Abstract

Steady-state cerebral blood flow (CBF) and dynamic cerebral autoregulation are reportedly maintained during −10° head-down tilt (HDT) despite slight increases in intracranial pressure (ICP). However, the higher ICP during −30° HDT may alter steady-state CBF and dynamic cerebral autoregulation. The present study hypothesized that steady-state CBF and dynamic cerebral autoregulation would be altered by higher ICP during −30° HDT than during 0° and −10° HDT. Seventeen healthy participants were positioned horizontal (0°) and in −10° HDT and −30° HDT for 10 min in random order on separate days. The arterial blood pressure waveform was obtained using a finger blood pressure device and the cerebral blood velocity waveform in the middle cerebral artery was obtained using transcranial Doppler sonography (TCD) for the last 6 min in each position. ICP was estimated using noninvasive ICP (nICP) based on TCD. Dynamic cerebral autoregulation was evaluated by spectral and transfer function analysis. Although nICP was significantly higher during −30° HDT (12.4 mmHg) than during −10° HDT (8.9 mmHg), no significant differences in steady-state mean cerebral blood velocity or transfer function gain in any frequency ranges were seen among all angles of HDT. Counter to our hypothesis, the present results suggest that steady-state CBF and dynamic cerebral autoregulation may be preserved during short-term −30° HDT despite the higher ICP compared with that during −10° HDT.

**NEW & NOTEWORTHY** This appears to be the first study to evaluate steady-state cerebral blood flow (CBF), dynamic cerebral autoregulation, and intracranial pressure (ICP) during −30° head-down tilt (HDT) compared with those during −10° HDT using noninvasive measurements. The results suggest that steady-state CBF and dynamic cerebral autoregulation are preserved despite the higher ICP during short-term −30° HDT compared with −10° HDT.

## Graphical abstract

**Figure:**
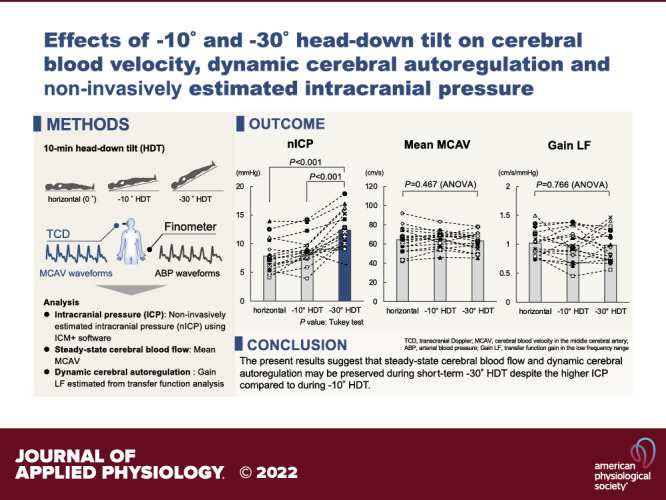

## INTRODUCTION

Alterations in cerebral circulation and intracranial pressure (ICP) induced by the cephalad fluid shift during spaceflight may be partially related to health problems in astronauts, such as headache (1) and space-associated neuro-ocular syndrome (SANS) (2). To better understand these important health issues in the field of space medicine, various studies focusing on cerebral circulation and ICP have been conducted in recent years (3, 4). However, the etiology of SANS remains contentious (2).

Both −6° and −10° head-down tilts (HDTs) have frequently been used as ground-based analogs of spaceflight to study the effects of cephalad fluid shift (5–7), although the effects on ICP may differ from those produced under real microgravity (8). The −10° HDT has been used to simulate the acute phase of exposure to microgravity, exaggerating the cephalad fluid shift (6, 7, 9, 10). Moreover, robotic-assisted laparoscopic surgeries have been performed in gynecology and urology, using −10° HDT or even steeper positions to better visualize the target organs (11, 12). In particular, pelvic robotic surgery can apply an approximately −30° head-down position (13). Various complications including vision problems have been reported after robotic-assisted laparoscopic surgeries with a steep head-down position (12). Furthermore, cases with neurological complications such as brain edema (14) or hemiparesis (15) have been reported after robotic-assisted laparoscopic surgery with −30° and −45° HDT. Increases in ICP and/or alterations in cerebral circulation during such a steep head-down position could be responsible for such surgical complications. A better understanding of the effects of −30° HDT on cerebral circulation is thus important.

Steady-state cerebral blood flow (CBF) and dynamic cerebral autoregulation that maintains CBF at a relatively constant level in response to “rapid changes” in ABP are reportedly preserved during −10° HDT (10, 16). Two previous studies have shown that cerebral blood velocity in the middle cerebral artery (MCAV) obtained by transcranial Doppler sonography (TCD) and dynamic cerebral autoregulation indices as evaluated by transfer function analysis did not change during −10° HDT (10, 16). On the other hand, ICP was reported to be slightly increased during short-term exposure to −10° HDT (8, 17). Lawley et al. (8) reported temporal changes in ICP during −6° HDT for 24 h and showed slight increases in ICP (+1.8 mmHg) in the first 5 min of HDT using direct measurement of ICP. Kurazumi et al. (17) also showed a slight increase in ICP (+1.9 mmHg) during −10° HDT for 10 min compared with the supine position using a noninvasive estimation method based on TCD. Steady-state CBF and dynamic cerebral autoregulation would thus be preserved during −10° HDT, despite slight increases in ICP. However, ICP should be further increased during −30° HDT, given the higher hydrostatic pressure effects of ICP, e.g., a linear relation to “sin (HDT angle)” at least from 0° to −20° HDT (18). Venostasis may further increase ICP via increases in the volume of intracranial venous blood, before complete compensation against the increases in total amount of brain tissue, cerebrospinal fluid, and cerebral blood are accomplished by replacement of CSF. Small changes in intracranial blood (e.g., a few milliliters) can affect ICP as noted in a previous study using HDT and lower body negative pressure (19). Conversely, the presence of internal jugular vein valves (20) may reduce hydrostatic pressure effects. In such cases, cerebral perfusion pressure (= mean arterial pressure at brain – ICP) may change and could alter steady-state CBF. Moreover, even if steady-state CBF remains largely unchanged, dynamic cerebral autoregulation could potentially still be altered by physiological changes under −30° HDT, similar to the effects seen under stable resting conditions in healthy subjects such as spaceflight (21) or hypoxia (22). However, the effects of −30° HDT on cerebral circulation including dynamic cerebral autoregulation have not been fully investigated.

We therefore conducted this study to test the hypothesis that −30° HDT would affect steady-state CBF and dynamic cerebral autoregulation, as confirmed by higher estimated ICP during −30° HDT compared with that during 0° HDT and −10° HDT. We evaluated dynamic cerebral autoregulation using transfer function analysis between MCAV obtained by TCD and arterial blood pressure (ABP) obtained by a finger blood pressure device during three HDT conditions (horizontal [i.e., 0° HDT], −10° HDT, or −30° HDT) for 10 min.

## METHODS

### Participants

Participants comprised 17 healthy volunteers (12 men, 5 women) with a mean [±standard deviation (SD)] age of 24 ± 2 yr, height of 169 ± 10 cm, and weight of 66 ± 17 kg. The study protocol was approved by the institutional review board at Nihon University School of Medicine (Approval no. 29-3-1) and was registered to the University Hospital Medical Information Network clinical trial registry (ID: UMIN000036541). All procedures adhered to the tenets of the Declaration of Helsinki. All participants provided written informed consent as well as a medical history regarding cardiovascular health and were screened based on a physical examination including electrocardiography (ECG) and blood pressure measurements. Exclusion criterion included failure to obtain MCAV by TCD. All experiments were performed ≥2 h after a meal. Participants were asked to refrain from heavy exercise or consumption of caffeinated or alcoholic beverages for at least 12 h before the experiments. All participants were familiarized with the measurement techniques and experimental conditions before the first data collection experiment. Participants were randomly exposed to all three angles of HDT [horizontal (0° HDT), −10° HDT, and −30° HDT] for 10 min at a similar time on different days (total, 3 days).

### Equipment

All experiments were performed in an environmentally controlled laboratory with an ambient temperature of 20–26°C and room air with environmental carbon dioxide <1,000 ppm. Participants lay supine on an electrical tilting bed, initially placed in the horizontal position. Three-lead ECG and pulse oximetry for arterial oxygen saturation (${\text{Sp}}_{{\text{O}}_{\text{2}}}$) were applied (Lifescope BSM-3800; Nihon Kohden, Tokyo, Japan). An infrared carbon dioxide sensor that can obtain capnograms via both mouth and nose was applied to detect respiratory rate and end-tidal carbon dioxide pressure ($ET_{CO_{2}}$; OLG-2800; Nihon Kohden). $ET_{CO_{2}}$ data were corrected for body temperature and ambient pressure, saturated with water vapor (37°C, 100% humidity). Arterial blood pressure at heart level (ABP_Heart_) was continuously measured in the left middle finger using a volume clamping method with photoplethysmography as part of the feedback loop used to maintain clamping (Finometer MIDI; Finapres Medical Systems, Amsterdam, the Netherlands) and a height-correction sensor was placed on the left arm at heart level (antecubital fossa along the anterior axillary line). To calibrate the ABP_Heart_, intermittent ABP using the oscillometric method with a cuff sphygmomanometer placed over the right brachial artery at heart level (antecubital fossa along the anterior axillary line) was measured before each data collection session (Lifescope BSM-3800; Nihon Kohden). MCAV was obtained continuously by TCD (EZ-Dop; Compumedics Germany GmbH, Sipplingen, Germany) at a depth of 50–60 mm using a 2-MHz probe, by the same experienced physician. The reproducibility of MCAV as measured by TCD is known to be good when performed by an experienced sonographer and careful attention is paid to probe placement (23). In the present study, the coefficient of variation in MCAV in the supine resting condition before the three angles of HDT was 5.5%, as achieved using the following procedure for probe fixation. The probe was fixed individually at the same position and at a constant angle using a customized molded probe holder made by the same experienced physician using an earplug and dental impression material to fit the facial bone and ear structures of each participant (3, 24). The same depth, power, and sample volume for each participant were used for all three angles of HDT. Waveforms of continuous ABP, MCAV, ECG, and capnogram were recorded at a sampling rate of 1 kHz using commercial software (Notocord-hem 3.3; Notocord, Paris, France) throughout the study.

### HDT Protocol

First, participants lay on an electric tilting bed (Minato Medical Science, Osaka, Japan) in a horizontal supine position. Following 15 min of rest, stabilizations of each measured waveform were confirmed for 6 min in that position. Participants were then tilted to one of the three following HDT angles for each experiment day, with the position maintained for 10 min: horizontal (0°), −10° HDT, and −30° HDT.

### Data Analysis

#### Steady-state hemodynamics and respiratory conditions.

Mean ABP from cuff sphygmomanometry immediately before each data collection point in the HDT position was used for steady-state mean ABP at the level of the heart (mean ABP_Heart_). Amplitude of the oscillations in mean ABP_Heart_ was calculated as maximum mean ABP_Heart_ minus minimum mean ABP_Heart_. Values for steady-state mean ABP at the level of the MCA (mean ABP_MCA_), steady-state mean MCAV, heart rate, respiratory rate and partial pressure of $ET_{CO_{2}}$ were obtained by averaging recorded waveforms for 6 min from 4 min after beginning HDT (4th–10th min of HDT). ${\text{Sp}}_{{\text{O}}_{\text{2}}}$ was manually recorded every minute and calculated by averaging the data.

To determine ABP at the level of the MCA (ABP_MCA_), the difference in hydrostatic pressure between the heart level (xiphoid process) and MCA level (external acoustic meatus) was added to the ABP_Heart_. The difference in hydrostatic pressure was calculated using manually measured distances between the levels of the heart and MCA, with the following formula:

Hydrostatic pressure difference (mmHg)=distance (cm) × 1.06/13.6 × 10

This is based on the assumption that the specific gravity of mercury at 37°C (density: 13,500 kg/m^3^) as compared with water at 37°C (density 993 kg/m^3^) is 13.6, and the specific gravity of whole blood at 37°C as compared with water at 37°C is 1.06 (25).

#### Spectral and transfer function analyses.

Beat-to-beat mean ABP_Heart_ and mean MCAV were obtained from recorded waveforms for 6 min from 4 min after beginning HDT (4th–10th min of HDT) by integrating signals within each cardiac cycle using the PC-based Notocord-Hem 3.3 software (Notocord). Beat-to-beat mean ABP_Heart_ and MCAV were linearly interpolated and resampled at 4 Hz for spectral and transfer function analyses. The time series of data were first subtracted by mean values. Fast Fourier transform and transfer function analyses were performed using a Hanning window on 512-point segments with 50% overlap. This process resulted in five segments of data recordings over 6 min. This analysis was based on international cerebral autoregulation research network-recommended algorithms (26). We analyzed data using DADiSP software (DSP Development, Cambridge, MA). The spectral power of mean ABP_Heart_ variability and mean MCAV variability, and transfer function gain, phase and coherence were calculated in the very low frequency (0.02–0.07 Hz), low frequency (0.07–0.20 Hz), and high frequency (0.20–0.35 Hz) ranges. These ranges were specifically based on the frequency-dependent property of dynamic cerebral autoregulation as previously proposed by transfer function analysis (27, 28). Coherence ranging between 0 and 1 reflects a linear relationship between mean ABP_Heart_ and mean MCAV variabilities. Transfer function gain represents the capability of distal cerebral arterioles to suppress transmission from mean ABP_Heart_ variability to mean MCAV variability. Larger gain implies that any given change in mean ABP_Heart_ leads to a larger change in mean MCAV and can be interpreted as attenuated dynamic cerebral autoregulation (28). The phase is the temporal relationship between mean ABP_Heart_ and mean MCAV variabilities.

#### Noninvasive ICP analysis.

The present study applied the mathematical model for estimating ICP described by Schmidt et al. (29). Previously, this model has been reported to correlate strongly with invasively measured ICP (30). This model is based on a concept of system analysis in which output signals (ICP) result from a systemic response to input signals (ABP) (29, 31). The system response is described in terms of a transfer function between ABP and ICP controlled by the relationship between ABP and CBF. Noninvasive ICP (nICP) was calculated by plugin “nICP Plugin” software (Klinkum Chemnitz gGmbH, Chemnitz, Germany) for ICM+ version 8.1 (Cambridge Enterprise: http://www.neurosurg.cam.ac.uk/icmplus/, Cambridge, UK). Analyses for nICP were performed as described in previous studies (17, 29). Briefly, waveforms for continuous ABP_MCA_ and MCAV recorded at 1 kHz were resampled at 100 Hz using Notocord-Hem 3.3 software (Notocord). Waveforms for ABP_MCA_ were calculated as described above. The last 6 min of each 10-min HDT was extracted for noninvasive ICP analysis. Calculations for nICP were performed for every 10-s window, and values of 36 windows were averaged.

In addition, noninvasive cerebral perfusion pressure (nCPP) was calculated by subtracting nICP from mean ABP_MCA_. Cerebrovascular resistance was expressed as the cerebrovascular resistance index (CVRi), where CVRi = nCPP/mean MCAV.

Data are presented as mean ± SD. Statistical analyses were performed using SigmaPlot version 14.0 software (Systat Software, San Jose, CA). Normal distributions of data were confirmed using the Shapiro–Wilk test. Physiological variables were compared using one-way repeated-measures analysis of variance (ANOVA) with angle of HDT [horizontal (0° HDT), −10° HDT, and −30° HDT], followed by Tukey’s testing for multiple comparisons. If variables did not follow a normal distribution, Friedman repeated measures analysis of variance on Ranks (Friedman) were performed, followed by the Tukey’s test on Ranks for multiple comparisons.

## RESULTS

All participants completed all HDT protocols. No participants complained of any symptoms such as headache or nausea throughout the experiments.

### nICP

Values for nICP were significantly higher as angle of HDT increased (ANOVA, *P* < 0.001; Table 1; Fig. 2*A*). Values of nICP were significantly higher during −30° HDT than during horizontal or −10° HDT (Tukey’s test, both *P* < 0.001).

**Table 1.** Indices of estimated intracranial pressure

	Horizontal	−10° HDT	−30° HDT	*P* Value	
nICP, mmHg	7.8 ± 2.8	8.9 ± 2.6	12.4 ± 3.4*†	<0.001	(A)

Values represent means ± SD. *P* values are obtained from one-way repeated-measures analysis of variance (A). *P < 0.001 compared with horizontal (Tukey’s test). †*P* < 0.001 compared with −10° HDT (Tukey’s test). HDT, head-down tilt; Horizontal, horizontal supine position (0°); nICP, noninvasive intracranial pressure.

### Steady-State Hemodynamic and Respiratory Condition

No significant differences in mean MCAV were seen among all angles of HDT (Table 2; Fig. 2*B*). Likewise, no significant differences in HR, mean ABP_Heart_, amplitude of mean ABP_heart_, or respiratory condition were evident among all angles of HDT. Only mean ABP_MCA_ was significantly higher in relation to increased angle of HDT (ANOVA, *P* < 0.001) and was significantly higher during −30° HDT than during horizontal or −10° HDT (Tukey’s test, both *P* < 0.001).

**Table 2.** Steady-state hemodynamics and respiratory condition

	Horizontal	−10° HDT	−30° HDT	*P* Value	
HR, beats/min	61 ± 9	60 ± 8	61 ± 8	0.748	(A)
Mean ABP_Heart_, mmHg	81 ± 6	80 ± 7	81 ± 8	0.520	(A)
Mean ABP_MCA_, mmHg	80 ± 6	82 ± 7	91 ± 8*†	<0.001	(A)
Amplitude of mean ABP_Heart_, mmHg	21 ± 7	20 ± 7	19 ± 8	0.382	(A)
Mean MCAV, cm/s	64 ± 13	65 ± 9	63 ± 10	0.467	(A)
nCPP, mmHg	72 ± 6	73 ± 6*	79 ± 8*§	<0.001	(A)
CVRi, mmHg/cm/s	1.16 ± 0.24	1.14 ± 0.17	1.28 ± 0.23#§	0.001	(A)
Resp-R, breaths/min	15 ± 3	15 ± 3	15 ± 4	0.821	(F)
${\text{Sp}}_{{\text{O}}_{\text{2}}}$, %	98 ± 1	98 ± 1	98 ± 1	0.598	(A)
$ET_{CO_{2}}$, Torr	38 ± 3	38 ± 2	38 ± 2	0.346	(A)

Values represent means ± SD. *P* values are obtained from one-way repeated-measures analysis of variance (A) or Friedman repeated-measures analysis of variance on ranks (F). *P < 0.001 compared with horizontal (Tukey’s test). †*P* < 0.001 compared with −10° HDT (Tukey’s test). #*P* < 0.01 compared with horizontal (Tukey’s test). §*P* < 0.01 compared with −10° HDT (Tukey’s test). CVRi, cerebrovascular resistance index; $ET_{CO_{2}}$, end-tidal carbon dioxide pressure; HDT, head-down tilt; Horizontal, horizontal supine position (0°); HR, heart rate; mean ABP_Heart_, mean arterial blood pressure at heart level; mean ABP_MCA_, mean arterial blood pressure at middle cerebral artery level; mean MCAV, mean cerebral blood velocity in the middle cerebral artery; nCPP, noninvasive cerebral perfusion pressure; Resp-R, respiratory rate; ${\text{Sp}}_{{\text{O}}_{\text{2}}}$, arterial oxygen saturation.

The nCPP increased significantly as angle of HDT increased (ANOVA, *P* < 0.001) and was significantly higher during −30° HDT than during horizontal and −10° HDT (Tukey’s test, *P* < 0.001 and *P* = 0.003, respectively). CVRi changed significantly (ANOVA, *P* < 0.001) and was significantly higher during −30° HDT than during horizontal or −10° HDT (Tukey’s test, *P* = 0.003 and *P* = 0.007, respectively).

### Spectral and Transfer Function Analyses

**Figure 1.**
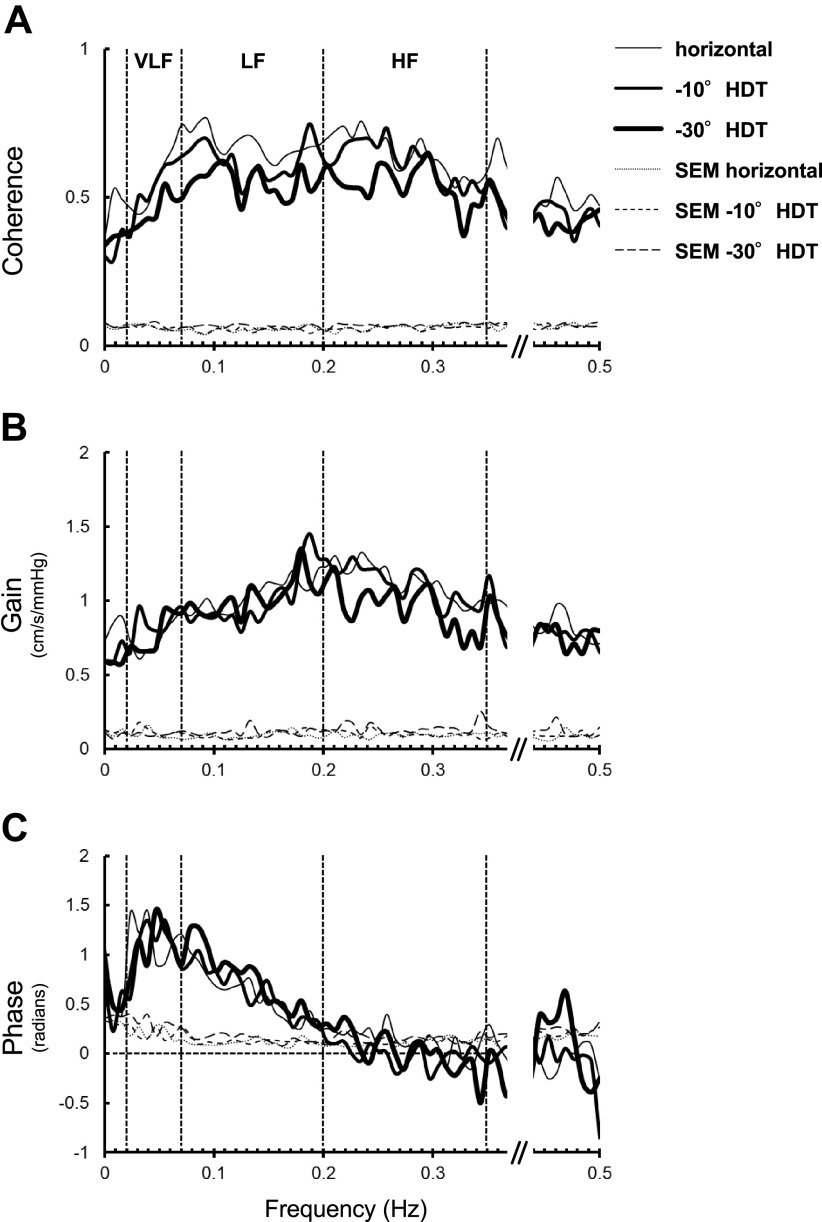
Group averages of transfer function analysis between mean arterial blood pressure variability and mean cerebral blood velocity variability in the middle cerebral artery during head-down tilt [horizontal (0° HDT), −10° HDT, and −30° HDT]. Coherence function (*A*), transfer function gain (*B*), and phase differences (*C*). HF, high frequency range (0.20–0.35 Hz); LF, low frequency range (0.07–0.20 Hz); VLF, very low frequency range (0.02–0.07 Hz), *n* = 17.

**Figure 2.**
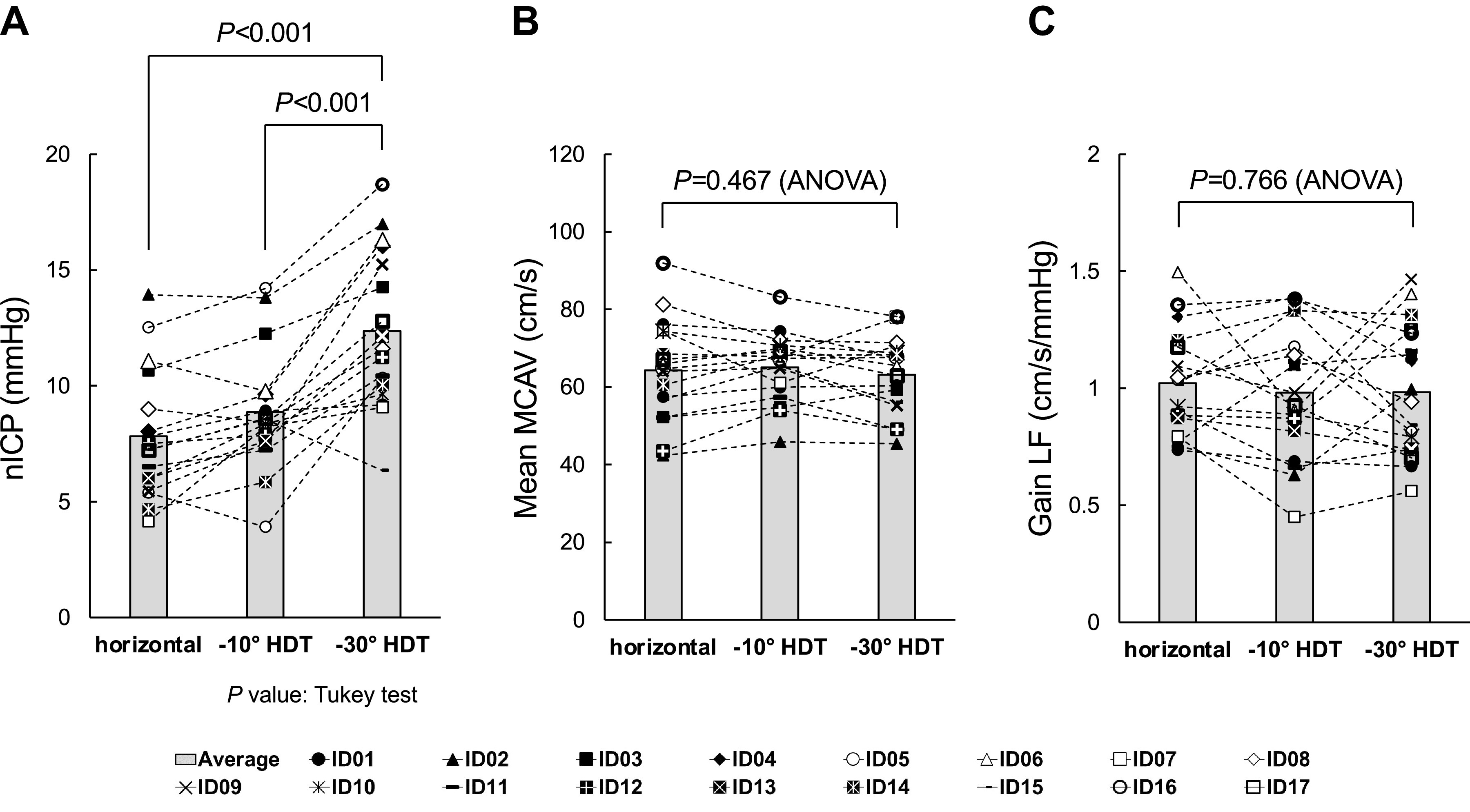
Group averages and individual changes in noninvasive intracranial pressure (nICP), mean cerebral blood velocity, and transfer function gain in the low-frequency range during head-down tilt [horizontal (0° HDT), −10° HDT, and −30° HDT]. nICP (*A*); mean cerebral blood velocity in the middle cerebral artery (mean MCAV; *B*); and transfer function gain in the low frequency range (Gain LF; *C*). One-way repeated-measures analysis of variance (ANOVA; *n* = 17; nICP, *P* < 0.001; mean MCAV, *P* = 0.467; Gain LF, *P* = 0.766) and the Tukey’s test were used to determine significant differences (nICP: horizontal vs. −30° HDT, *P* < 0.001; −10° HDT vs. −30° HDT, *P* < 0.001).

No significant differences in transfer function gain were seen in any frequency ranges among all angles of HDT (Table 3; Figs. 1 and 2*C*). Significant differences were only seen in the spectral power of mean MCAV variability in the low frequency range (Friedman, *P* = 0.011), coherence in the low and high frequency ranges (ANOVA, *P* = 0.003 and *P* = 0.028, respectively), and phase in the high frequency range (Friedman, *P* = 0.023). The spectral power of mean MCAV variability in the low frequency range was significantly lower during −30° HDT than during horizontal or −10° HDT (Tukey’s test, *P* = 0.017 and *P* = 0.043, respectively). Coherence in the low and high frequency ranges was significantly lower during 30° HDT than during horizontal (Tukey’s test; *P* = 0.002 and *P* = 0.028, respectively). Phase in the high frequency range was significantly lower during −10° HDT than during −30° HDT (Tukey’s test, *P* = 0.017).

**Table 3.** Spectral and transfer function analyses

	Horizontal	−10° HDT	−30° HDT	*P* Value	
VLF MAPv, mmHg^2^	6.61 ± 6.76	7.52 ± 10.01	5.03 ± 6.08	0.059	(F)
VLF MCAVv, cm^2^/s^2^	6.66 ± 6.42	6.82 ± 6.42	4.49 ± 3.98	0.101	(F)
Coherence VLF	0.53 ± 0.18	0.52 ± 0.17	0.45 ± 0.22	0.196	(A)
Gain VLF, cm/s/mmHg	0.72 ± 0.31	0.82 ± 0.32	0.72 ± 0.25	0.242	(A)
Phase VLF, rad	1.17 ± 0.47	1.10 ± 0.47	1.10 ± 0.62	0.892	(A)
LF MAPv, mmHg^2^	2.19 ± 2.37	1.96 ± 2.24	1.40 ± 1.52	0.102	(A)
LF MCAVv, cm^2^/s^2^	2.86 ± 3.28	2.13 ± 1.88	1.95 ± 2.07#$	0.011	(F)
Coherence LF	0.68 ± 0.12	0.62 ± 0.12	0.55 ± 0.15§	0.003	(A)
Gain LF, cm/s/mmHg	1.02 ± 0.23	0.98 ± 0.28	0.98 ± 0.28	0.766	(A)
Phase LF, rad	0.64 ± 0.25	0.66 ± 0.29	0.78 ± 0.36	0.113	(F)
HF MAPv, mmHg^2^	0.19 ± 0.21	0.14 ± 0.12	0.13 ± 0.14	0.161	(F)
HF MCAVv, cm^2^/s^2^	0.33 ± 0.27	0.27 ± 0.20	0.25 ± 0.20	0.465	(F)
Coherence HF	0.65 ± 0.15	0.62 ± 0.14	0.54 ± 0.19#	0.028	(A)
Gain HF, cm/s/mmHg	1.14 ± 0.22	1.12 ± 0.25	1.02 ± 0.24	0.178	(A)
Phase HF, rad	0.13 ± 0.22	0.00 ± 0.25#	0.04 ± 0.28	0.023	(F)

Values represent means ± SD. *P* values are obtained from one-way repeated-measures analysis of variance (A) or Friedman repeated-measures analysis of variance on ranks (F). #*P* < 0.05 compared with horizontal (Tukey’s test). §*P* < 0.01 compared with horizontal (Tukey’s test). $*P* < 0.05 compared with −10° HDT (Tukey’s test). gain, transfer function gain; HDT, head-down tilt; HF, high frequency range (0.20–0.35 Hz); Horizontal, horizontal supine position (0°); LF, low frequency range (0.07–0.20 Hz); MAPv, mean arterial blood pressure variability; MCAVv, mean cerebral blood velocity variability in the middle cerebral artery; VLF, very low-frequency range (0.02–0.07 Hz).

## DISCUSSION

The present study found no significant differences in mean MCAV and transfer function gain among all angles of HDT, despite confirming the expected obvious increases in estimated ICP during −30° HDT. These results suggest that steady-state CBF and dynamic cerebral autoregulation remained unaltered during short-term −30° HDT despite the increased ICP.

### ICP

An increase in cerebral venous pressure by hydrostatic pressure effects should be primarily responsible for increases in ICP with increasing angles of HDT. The internal jugular veins are the main routes of venous drainage and stay open in the low head position (32, 33). Given an open venous system during HDT positions, increases in cerebral venous pressure in the sagittal sinus with hydrostatic pressure gradients can directly increase ICP. By this mechanism, ICP may increase in an almost-linear fashion with increasing hydrostatic pressure during HDT. However, the increases in ICP during HDT might also be enhanced by increases in cerebral venous blood, particularly during steeper HDT that may even lower the very low compliance of the intracranial compartment. On the other hand, some individuals possess internal jugular vein valves (20) that would reduce the hydrostatic pressure effects on cerebral venous pressure or regurgitations in the internal jugular vein, although the present study did not check the presence of valves.

### Steady-State CBF

In the present study, no difference in mean MCAV was identified among the three angles of HDT. Thus, counter to our hypothesis, the present results suggest that steady-state CBF may be preserved during steep HDT, despite the higher ICP during steep HDT.

Previous reports have found that steady-state CBF was preserved during −10° HDT for 5–10 min (10, 16) consistent with the present results of no change in mean MCAV during −10° HDT. In addition, Ogoh et al. reported no changes in internal carotid artery blood flow during −10° and −20° HDT for 10 min (34). The present study showed no change in steady-state CBF during −30° HDT for 10 min. Furthermore, previous studies have reported no marked changes in CBF during 10 min of either −45° or −90° HDT (35, 36). Taken together, the cerebrovasculature in healthy subjects seems remarkably robust and is able to maintain steady-state CBF during short-term extreme HDT. On the other hand, Marshall-Goebel et al. reported decreased CBF after 4.5 h of HDT with three angles (−6°, −12°, −18°) using phase-contrast magnetic resonance imaging (37). Montero et al. showed a tendency toward decreased CBF in the MCA by 3-h steep HDT (−30°) using TCD (38). Therefore, maintenance of steady-state CBF during −30° HDT may hold true only for short-term exposures.

### Dynamic Cerebral Autoregulation

We had hypothesized that dynamic cerebral autoregulation would be altered by the higher ICP during −30° HDT than during −10° HDT. However, counter to our hypothesis, no differences in group averages of transfer function gain were seen for any frequency ranges between −10° HDT and −30° HDT in the present study. These results can be interpreted as no difference of magnitude of transmission from ABP oscillation to CBF fluctuation between −10° HDT and −30° HDT. The present findings therefore suggest that dynamic cerebral autoregulation may be preserved during −30° HDT, as well as in steady-state CBF.

At least three possibilities may explain the preserved steady-state CBF and dynamic cerebral autoregulation during −30° HDT. First, ICP during −30° HDT may not have been sufficiently high to affect dynamic cerebral autoregulation. Previous studies have shown that ICP ≥ 20 mmHg is associated with impaired dynamic cerebral autoregulation (39, 40). Although nICP was significantly higher during −30° HDT than during −10° HDT, as expected, absolute nICP during −30° HDT (12.4 mmHg) was lower than the value at 20 mmHg. Second, dynamic cerebral autoregulation in healthy people must represent a more robust system than that in patients and may not be affected by increased ICP from −30° HDT. Third, the changes in dynamic cerebral autoregulation may depend on the duration of HDT, since the duration in the present study was very short. In addition to these three factors (i.e., level of increase in ICP, relative health of subjects, and duration), for cases with complications after robotic-assisted laparoscopic surgery at HDT, combined effects with hypercapnia might be more important than HDT alone (40). Therefore, the present results cannot reject relationships between altered cerebral autoregulation and complications after such surgeries at HDT. These factors need to be investigated in the future studies.

A low signal-to-noise ratio with decreasing input and output signals may be mainly responsible for the decreased coherence in the low and high frequency ranges during −30° HDT. The present study showed that coherence in the low and high frequency ranges during −30° HDT was decreased significantly compared with horizontal. In general, two mechanisms for decreased coherence are considered: *1*) presence of a low signal-to-noise ratio with increased noise and/or decreased signals and *2*) nonlinearity between change in pressure (input signal) and velocity (output signal), including effective dynamic cerebral autoregulation or the presence of other determinants of cerebral blood flow variability. In the present study, mean ABP variability in the low frequency range tended to decrease and mean MCAV variability in the low frequency range decreased significantly with increasing angles of HDT. A similar tendency was observed in the high frequency range. We therefore cannot exclude the possibility that decreases to both input signal (mean ABP variability) and output signal (mean MCAV variability) induced relative decreases in signal-to-noise ratio, resulting in decreased coherence. The reduction in coherence could also indicate enhanced dynamic cerebral autoregulation, although this possibility seems unlikely given the lack of changes in transfer function gain or phase in the low frequency range. In addition, cerebral vasoconstriction and/or changes in cerebral vasomotor sympathetic nervous activity could potentially affect mean MCAV variability more than mean ABP variability, reducing coherence between mean MCAV and mean ABP variabilities during −30° HDT. In fact, CVRi increased significantly during −30° HDT.

#### Limitations.

Three major limitations should be considered in the present study. The most obvious potential limitation of the present study is that dynamic cerebral autoregulation and ICP were estimated in a noninvasive manner from MCAV obtained by TCD and ABP obtained by Finometer. This is because assessment of CBF using MCAV obtained by TCD is based on an assumption of a relatively constant diameter of the MCA. Although previous studies have confirmed that changes in MCAV are actually proportional to global CBF as measured on single photon emission computed tomography (SPECT) (41) or the ^131^Xe clearance technique (42), or internal carotid artery flow with an electromagnetic flowmeter (43), we cannot exclude the possibility that MCA diameter was altered during −30° HDT. Also, the shape of the ABP waveform obtained by Finometer could differ from those obtained invasively from the radial or femoral artery. Results based on these techniques should thus be interpreted with caution.

Second, decreased ABP variability by −30° HDT could deteriorate the accuracy of transfer function gain due to lower inputs, as described above. Lower power of ABP variability has been reported to lead to low reproducibility of dynamic cerebral autoregulation using transfer function analysis (44, 45).

Lastly, the noninvasive ICP estimation used in the present study displays three differences from the original ICP estimation method (noninvasive versus invasive ABP measurement, head-down position versus horizontal position, and healthy people versus traumatic brain injury patients). These differences might contribute to less-accurate ICP estimation in the present study. The original method for estimating nICP is based on invasive ABP waveforms in the radial or femoral artery (29). Such differences might in turn affect estimations of nICP derived from ABP waveform analysis. Also, the head-down position might enlarge the dissociation of shape and/or absolute value between true ABP in the MCA and ABP obtained from the Finometer. In addition, original evaluation methods have been developed based on data from patients with traumatic brain injury, unlike the present study in which the participants were healthy volunteers. Estimations of ICP may thus have been less accurate for healthy individuals. These may lead to nonsignificant differences in estimated ICP between horizontal and −10° HDT in the present study.

### Conclusion

As expected, ICP as estimated using noninvasive measurements was higher during −30° HDT than during horizontal or −10° HDT in the present study. However, counter to our hypothesis, no significant differences in mean MCAV or transfer function gain were seen among all angles of HDT. These results suggest that steady-state CBF and dynamic cerebral autoregulation may be preserved during short-term −30° HDT despite the higher ICP compared with that during −10° HDT.

## GRANTS

This study was supported by JSPS KAKENHI (Grant No. JP15H05939) for conducting the experiments, and by JSPS KAKENHI (Grant No. JP20K06844) for analyzing the data and editing the manuscript.

## DISCLOSURES

No conflicts of interest, financial or otherwise, are declared by the authors.

## AUTHOR CONTRIBUTIONS

T.Ka. and K.I. conceived and designed research; T.Ka., T.Ku., T.Ko., C.T., Y.O., and K.I. performed experiments; T.Ka. and K.I. analyzed data; T.Ka., T.Ku., T.Ko., C.T., Y.O., and K.I. interpreted results of experiments; T.Ka. prepared figures; T.Ka. and K.I. drafted manuscript; T.Ka., T.Ku., T.Ko, C.T., Y.O., and K.I. edited and revised manuscript; T.Ka., T.Ku., T.Ko., C.T., Y.O., and K.I. approved final version of manuscript.

## Figures and Tables

**Figure A1.**
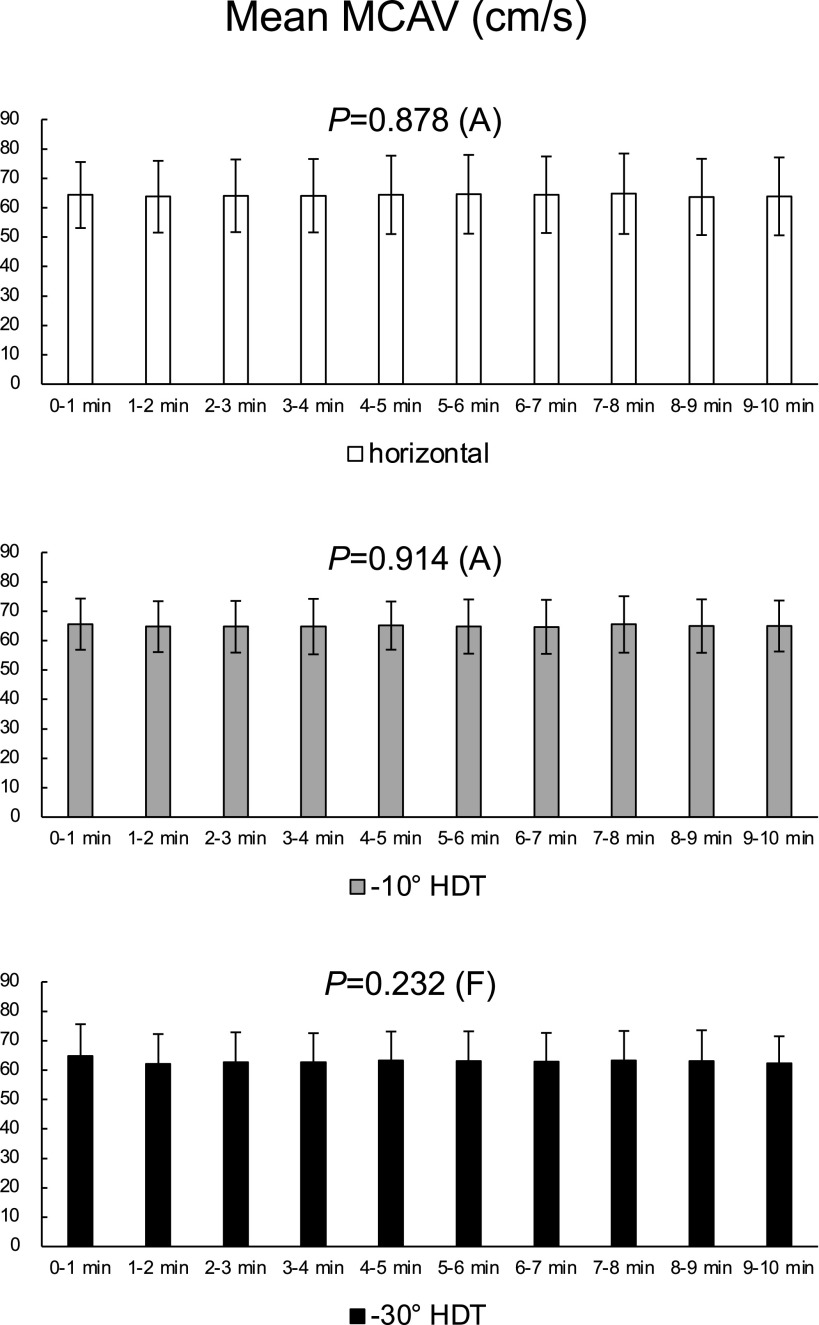
Time course of changes in 1-min average of mean cerebral blood velocity in the middle cerebral artery (MCAV) during three angles of head-down tilt (horizontal [0° HDT], −10° HDT, and −30°HDT). Values represent means ± SD [*n* = 17 (12 men, 5 women)]. The *P* values are obtained from one-way repeated-measures analysis of variance (A) or Friedman repeated-measures analysis of variance on ranks (F).

## APPENDIX

Figure A1 shows 1-min averages for MCAV at all angles of head-down tilt [horizontal (0° HDT), −10° HDT, and −30°HDT]. MCAV did not change significantly during any angles of 10-min HDT.

## References

[1] Vein AA, Koppen H, Haan J, Terwindt GM, Ferrari MD. Space headache: a new secondary headache. Cephalalgia 29: 683–686, 2009. doi:10.1111/j.1468-2982.2008.01775.x. 19175610

[2] Lee AG, Mader TH, Gibson CR, Tarver W, Rabiei P, Riascos RF, Galdamez LA, Brunstetter T. Author correction: spaceflight associated neuro-ocular syndrome (SANS) and the neuro-ophthalmologic effects of microgravity: a review and an update. NPJ Microgravity 6: 23, 2020. doi:10.1038/s41526-020-00114-8. 32047839 PMC7005826

[3] Iwasaki KI, Ogawa Y, Kurazumi T, Imaduddin SM, Mukai C, Furukawa S, Yanagida R, Kato T, Konishi T, Shinojima A, Levine BD, Heldt T. Long-duration spaceflight alters estimated intracranial pressure and cerebral blood velocity. J Physiol 599: 1067–1081, 2021. doi:10.1113/JP280318. 33103234 PMC7894300

[4] Marshall-Goebel K, Damani R, Bershad EM. Brain physiological response and adaptation during spaceflight. Neurosurgery 85: E815–E821, 2019. doi:10.1093/neuros/nyz203. 31215633

[5] Kermorgant M, Nasr N, Czosnyka M, Arvanitis DN, Hélissen O, Senard JM, Pavy-Le Traon A. Impacts of microgravity analogs to spaceflight on cerebral autoregulation. Front Physiol 11: 778, 2020. doi:10.3389/fphys.2020.00778. 32719617 PMC7350784

[6] Gharib C, Gauquelin G, Pequignot JM, Geelen G, Bizollon CA, Guell A. Early hormonal effects of head-down tilt (-10 degrees) in humans. Aviat Space Environ Med 59: 624–629, 1988. 3408423

[7] Mader TH, Taylor GR, Hunter N, Caputo M, Meehan RT. Intraocular pressure, retinal vascular, and visual acuity changes during 48 hours of 10 degrees head-down tilt. Aviat Space Environ Med 61: 810–813, 1990. 2241746

[8] Lawley JS, Petersen LG, Howden EJ, Sarma S, Cornwell WK, Zhang R, Whitworth LA, Williams MA, Levine BD. Effect of gravity and microgravity on intracranial pressure. J Physiol 595: 2115–2127, 2017. doi:10.1113/JP273557. 28092926 PMC5350445

[9] Frey MA, Mader TH, Bagian JP, Charles JB, Meehan RT. Cerebral blood velocity and other cardiovascular responses to 2 days of head-down tilt. J Appl Physiol (1985) 74: 319–325, 1993. doi:10.1152/jappl.1993.74.1.319. 8444709

[10] Kurazumi T, Ogawa Y, Yanagida R, Morisaki H, Iwasaki KI. Dynamic cerebral autoregulation during the combination of mild hypercapnia and cephalad fluid shift. Aerosp Med Hum Perform 88: 819–826, 2017. doi:10.3357/AMHP.4870.2017. 28818140

[11] Ghomi A, Kramer C, Askari R, Chavan NR, Einarsson JI. Trendelenburg position in gynecologic robotic-assisted surgery. J Minim Invasive Gynecol 19: 485–489, 2012. doi:10.1016/j.jmig.2012.03.019. 22748954

[12] Souki FG, Rodriguez-Blanco YF, Polu SR, Eber S, Candiotti KA. Survey of anesthesiologists' practices related to steep Trendelenburg positioning in the USA. BMC Anesthesiol 18: 117, 2018. doi:10.1186/s12871-018-0578-5. 30131061 PMC6104011

[13] Gould C, Cull T, Wu YX, Osmundsen B. Blinded measure of Trendelenburg angle in pelvic robotic surgery. J Minim Invasive Gynecol 19: 465–468, 2012. doi:10.1016/j.jmig.2012.03.014. 22621993

[14] Barr C, Madhuri TK, Prabhu P, Butler-Manuel S, Tailor A. Cerebral oedema following robotic surgery: a rare complication. Arch Gynecol Obstet 290: 1041–1044, 2014. doi:10.1007/s00404-014-3355-9. 25096953

[15] Pandey R, Garg R, Darlong V, Punj J, Chandralekha. Hemiparesis after robotic laparoscopic radical cystectomy and ileal conduit formation in steep Trendelenburg position. J Robot Surg 6: 269–271, 2012. doi:10.1007/s11701-011-0302-7. 27638287

[16] Cooke WH, Pellegrini GL, Kovalenko OA. Dynamic cerebral autoregulation is preserved during acute head-down tilt. J Appl Physiol (1985) 95: 1439–1445, 2003. doi:10.1152/japplphysiol.00524.2003. 12832430

[17] Kurazumi T, Ogawa Y, Yanagida R, Morisaki H, Iwasaki KI. Non-invasive intracranial pressure estimation during combined exposure to CO_2_ and head-down tilt. Aerosp Med Hum Perform 89: 365–370, 2018. doi:10.3357/AMHP.5015.2018. 29562966

[18] Petersen LG, Petersen JC, Andresen M, Secher NH, Juhler M. Postural influence on intracranial and cerebral perfusion pressure in ambulatory neurosurgical patients. Am J Physiol Regul Integr Comp Physiol 310: R100–R104, 2016. doi:10.1152/ajpregu.00302.2015. 26468260

[19] Petersen LG, Lawley JS, Lilja‐Cyron A, Petersen JCG, Howden EJ, Sarma S, Cornwell WK 3rd, Zhang R, Whitworth LA, Williams MA, Juhler M, Levine BD. Lower body negative pressure to safely reduce intracranial pressure. J Physiol 597: 237–248, 2019. doi:10.1113/JP276557. 30286250 PMC6312426

[20] Menegatti E, Tessari M, Gianesini S, Vannini ME, Sisini F, Zamboni P. Human internal jugular valve M-mode ultrasound characterization. Curr Neurovasc Res 11: 149–155, 2014. doi:10.2174/1567202611666140408094014. 24712644 PMC4031920

[21] Iwasaki K, Levine BD, Zhang R, Zuckerman JH, Pawelczyk JA, Diedrich A, Ertl AC, Cox JF, Cooke WH, Giller CA, Ray CA, Lane LD, Buckey JC Jr, Baisch FJ, Eckberg DL, Robertson D, Biaggioni I, Blomqvist CG. Human cerebral autoregulation before, during and after spaceflight. J Physiol 579: 799–810, 2007. doi:10.1113/jphysiol.2006.119636. 17185344 PMC2151354

[22] Iwasaki K, Ogawa Y, Shibata S, Aoki K. Acute exposure to normobaric mild hypoxia alters dynamic relationships between blood pressure and cerebral blood flow at very low frequency. J Cereb Blood Flow Metab 27: 776–784, 2007. doi:10.1038/sj.jcbfm.9600384. 16926845

[23] Brodie FG, Atkins ER, Robinson TG, Panerai RB. Reliability of dynamic cerebral autoregulation measurement using spontaneous fluctuations in blood pressure. Clin Sci (Lond) 116: 513–520, 2009. doi:10.1042/CS20080236. 18939945

[24] Giller CA, Giller AM. A new method for fixation of probes for transcranial Doppler ultrasound. J Neuroimaging 7: 103–105, 1997. doi:10.1111/jon199772103. 9128449

[25] Trudnowski RJ, Rico RC. Specific gravity of blood and plasma at 4 and 37 degrees C. Clin Chem 20: 615–616, 1974. doi:10.1093/clinchem/20.5.615. 4826961

[26] Claassen JA, Meel-van den Abeelen AS, Simpson DM, Panerai RB; International Cerebral Autoregulation Research Network (CARNet). Transfer function analysis of dynamic cerebral autoregulation: a white paper from the International Cerebral Autoregulation Research Network. J Cereb Blood Flow Metab 36: 665–680, 2016. doi:10.1177/0271678X15626425. 26782760 PMC4821028

[27] Giller CA. The frequency-dependent behavior of cerebral autoregulation. Neurosurgery 27: 362–368, 1990. doi:10.1097/00006123-199009000-00004. 2234328

[28] Zhang R, Zuckerman JH, Giller CA, Levine BD. Transfer function analysis of dynamic cerebral autoregulation in humans. Am J Physiol Heart Circ Physiol 274: H233–H241, 1998. doi:10.1152/ajpheart.1998.274.1.h233. 9458872

[29] Schmidt B, Klingelhöfer J, Schwarze JJ, Sander D, Wittich I. Noninvasive prediction of intracranial pressure curves using transcranial Doppler ultrasonography and blood pressure curves. Stroke 28: 2465–2472, 1997. doi:10.1161/01.str.28.12.2465. 9412634

[30] Cardim D, Robba C, Donnelly J, Bohdanowicz M, Schmidt B, Damian M, Varsos GV, Liu X, Cabeleira M, Frigieri G, Cabella B, Smielewski P, Mascarenhas S, Czosnyka M. Prospective study on noninvasive assessment of intracranial pressure in traumatic brain-injured patients: comparison of four methods. J Neurotrauma 33: 792–802, 2016. doi:10.1089/neu.2015.4134. 26414916 PMC4841086

[31] Kasuga Y, Nagai H, Hasegawa Y, Nitta M. Transmission characteristics of pulse waves in the intracranial cavity of dogs. J Neurosurg 66: 907–914, 1987. doi:10.3171/jns.1987.66.6.0907. 3572519

[32] Gisolf J, van Lieshout JJ, van Heusden K, Pott F, Stok WJ, Karemaker JM. Human cerebral venous outflow pathway depends on posture and central venous pressure. J Physiol 560: 317–327, 2004. doi:10.1113/jphysiol.2004.070409. 15284348 PMC1665206

[33] Valdueza JM, von Münster T, Hoffman O, Schreiber S, Einhäupl KM. Postural dependency of the cerebral venous outflow. Lancet 355: 200–201, 2000. doi:10.1016/s0140-6736(99)04804-7. 10675123

[34] Ogoh S, Washio T, Paton JFR, Fisher JP, Petersen LG. Gravitational effects on intracranial pressure and blood flow regulation in young men: a potential shunting role for the external carotid artery. J Appl Physiol (1985) 129: 901–908, 2020. doi:10.1152/japplphysiol.00369.2020. 32816640

[35] Gelinas JC, Marsden KR, Tzeng YC, Smirl JD, Smith KJ, Willie CK, Lewis NC, Binsted G, Bailey DM, Bakker A, Day TA, Ainslie PN. Influence of posture on the regulation of cerebral perfusion. Aviat Space Environ Med 83: 751–757, 2012. doi:10.3357/asem.3269.2012. 22872988

[36] Tymko MM, Skow RJ, MacKay CM, Day TA. Steady-state tilt has no effect on cerebrovascular CO2 reactivity in anterior and posterior cerebral circulations. Exp Physiol 100: 839–851, 2015. doi:10.1113/EP085084. 25966669

[37] Marshall–Goebel K, Ambarki K, Eklund A, Malm J, Mulder E, Gerlach D, Bershad E, Rittweger J. Effects of short-term exposure to head-down tilt on cerebral hemodynamics: a prospective evaluation of a spaceflight analog using phase-contrast MRI. J Appl Physiol (1985) 120: 1466–1473, 2016. doi:10.1152/japplphysiol.00841. 27013606 PMC4909835

[38] Montero D, Rauber S. Brain perfusion and arterial blood flow velocity during prolonged body tilting. Aerosp Med Hum Perform 87: 682–687, 2016. doi:10.3357/AMHP.4546.2016. 27634602

[39] Panerai RB, Hudson V, Fan L, Mahony P, Yeoman PM, Hope T, Evans DH. Assessment of dynamic cerebral autoregulation based on spontaneous fluctuations in arterial blood pressure and intracranial pressure. Physiol Meas 23: 59–72, 2002. doi:10.1088/0967-3334/23/1/306. 11876242

[40] de-Lima-Oliveira M, Salinet AM, Nogueira RC, de Azevedo DS, Paiva WS, Teixeira MJ, Bor-Seng-Shu E. Intracranial hypertension and cerebral autoregulation: a systematic review and meta-analysis. World Neurosurg 113: 110–124, 2018. doi:10.1016/j.wneu.2018.01.194. 29421451

[41] Larsen FS, Olsen KS, Hansen BA, Paulson OB, Knudsen GM. Transcranial Doppler is valid for determination of the lower limit of cerebral blood flow autoregulation. Stroke 25: 1985–1988, 1994. doi:10.1161/01.str.25.10.1985. 7916502

[42] Bishop CC, Powell S, Rutt D, Browse NL. Transcranial Doppler measurement of middle cerebral artery blood flow velocity: a validation study. Stroke 17: 913–915, 1986. doi:10.1161/01.str.17.5.913. 3764963

[43] Newell DW, Aaslid R, Lam A, Mayberg TS, Winn HR. Comparison of flow and velocity during dynamic autoregulation testing in humans. Stroke 25: 793–797, 1994. doi:10.1161/01.str.25.4.793. 7909175

[44] Claassen JA, Levine BD, Zhang R. Dynamic cerebral autoregulation during repeated squat-stand maneuvers. J Appl Physiol (1985) 106: 153–160, 2009. doi:10.1152/japplphysiol.90822.2008. 18974368 PMC2636935

[45] Elting JW, Sanders ML, Panerai RB, Aries M, Bor-Seng-Shu E, Caicedo A, Chacon M, Gommer ED, Van Huffel S, Jara JL, Kostoglou K, Mahdi A, Marmarelis VZ, Mitsis GD, Müller M, Nikolic D, Nogueira RC, Payne SJ, Puppo C, Shin DC, Simpson DM, Tarumi T, Yelicich B, Zhang R, Claassen JAHR. Assessment of dynamic cerebral autoregulation in humans: is reproducibility dependent on blood pressure variability? PLoS One 15: e0227651, 2020. doi:10.1371/journal.pone.0227651. 31923919 PMC6954074

